# Clinically interpretable deep learning for breast cancer missense variant pathogenicity prediction

**DOI:** 10.3389/fbinf.2026.1844071

**Published:** 2026-07-21

**Authors:** Rahaf M. Ahmad, Noura AlDhaheri, Mohd Saberi Mohamad, Bassam R. Ali

**Affiliations:** 1 Department of Genetics and Genomics, College of Medical and Health Sciences, United Arab Emirates University, Al-Ain, United Arab Emirates; 2 Centre for Advanced Analytics, CEO for Artificial Intelligence, Faculty of Engineering and Technology, Multimedia University, Melaka, Malaysia; 3 Department of Biosystems Engineering, Faculty of Agricultural Technology, Universitas Brawijaya, Malang, Indonesia; 4 Zayed Center for Heath Sciences, College of Medicine and Health Sciences, United Arab Emirates University, Al-Ain, United Arab Emirates

**Keywords:** breast cancer, clinical interpretability, deep learning, missense variants, pathogenicity prediction

## Abstract

**Background:**

Missense variants in breast cancer remain diagnostically challenging due to their functional diversity and complex genomic contexts. Conventional laboratory assays for evaluating pathogenicity are labor-intensive, costly, and often impractical for large-scale screening, creating a pressing need for accurate, scalable, and clinically interpretable computational approaches.

**Methods:**

In this study, we present a novel deep learning framework for predicting the pathogenicity of breast cancer missense variants, integrating comprehensive preprocessing, advanced imputation, rigorous model benchmarking, and explainability. Genetic variants were curated from multiple genomic databases, annotated using the Ensembl Variant Effect Predictor (VEP), and processed with Variational Autoencoders (VAE) for missing-value imputation. Seven deep learning models, MLP, CNN, DNN, RNN, LSTM, GRU, and Transformer, were trained and evaluated across 11 performance metrics. To quantify performance stability, each model was trained across five random seeds; mean AUC ± SD across seeds is reported as the primary performance estimate, with the best-seed run used only for LIME and PMI interpretability analyses. Recursive feature elimination, permutation importance (PMI), and Local Interpretable Model-Agnostic Explanations (LIME) were employed to enhance transparency. Statistical analyses, including Z-tests, ANOVA, and calibration assessments, validated performance consistency and inter-model differences.

**Results:**

GRU achieved the highest internal AUC (0.9956 [95% CI 0.9936–0.9972]; mean across five seeds 0.9941 ± 0.0011), with precision 0.9967 and calibration ECE 0.0095. Externally, LSTM led with AUC 0.9457, exceeding all eleven standalone predictors benchmarked on the same set. Models showed strong alignment with conservation signals such as phyloP470way and Eigen-PC scores. Notably, the pipeline provides performance metrics with 95% confidence intervals and incorporates case-level LIME visualizations for true positive, true negative, false positive, and false negative predictions, bolstering interpretability and clinical relevance.

**Conclusion:**

This work delivers one of the most comprehensive evaluations of deep learning in breast cancer variant classification to date. By combining high-performance sequential models with interpretable AI tools, the proposed framework provides a reproducible, transparent benchmark for variant pathogenicity prediction and a foundation for future research use and translation in cancer genomics.

## Introduction

Breast cancer remains a leading cause of cancer-related mortality in women globally, characterized by its molecular and genetic heterogeneity (Lefebvre et al., 2016). Among the array of genetic mutations implicated in its development, missense variants hold particular clinical relevance (Rony et al., 2025). These mutations result in single amino acid substitutions that can either be pathogenic by disrupting the protein function or remain benign, necessitating accurate methods to predict their pathogenicity (Dorling et al., 2022). Traditional laboratory assays for evaluating missense variants are often time-consuming and expensive, prompting a growing reliance on computational approaches. Deep learning (DL), a subfield of artificial intelligence, has emerged as a transformative force in precision oncology, offering novel methodologies to decode complex biological data and forecast the pathogenic potential of genetic variants (Shiri et al., 2023; Aburass et al., 2024). Deep learning models, such as Convolutional Neural Networks (CNNs), Recurrent Neural Networks (RNNs), Deep Neural Networks (DNNs), Long Short-Term Memory networks (LSTMs), Gated Recurrent Units (GRUs), Transformers, and Multilayer Perceptrons (MLPs), each offer unique architectural features that make them well-suited to variant interpretation. CNNs are adept at recognizing spatial hierarchies, RNNs and their derivatives excel at modeling sequential data, and Transformers offer unparalleled capabilities in capturing global dependencies through attention mechanisms (Shafi and Chinnappan, 2024). The integration of these models into genomics has enabled improved classification of missense variants, advancing personalized diagnostic strategies and therapeutic interventions (Rehana et al., 2023). CNNs have traditionally demonstrated strong performance in image-based classification tasks but have been adapted effectively for genomics, particularly in analyzing DNA sequences and structural representations of proteins. CNNs apply convolutional filters to detect spatial patterns, a property utilized to identify sequence motifs indicative of variant pathogenicity. RNNs, and especially their advanced forms such as LSTMs and GRUs, are optimized for sequential data (Aburass et al., 2024; Krikid et al., 2024). These models maintain context over time, making them ideal for modeling nucleotide sequences where each base’s impact may depend on its position within the sequence. While traditional RNNs suffer from vanishing gradients, LSTM and GRU architectures overcome this limitation by employing gated mechanisms to retain long-range dependencies. Elsamahy’s group highlighted the comparative strength of LSTM and GRU models in handling sequential variant data, with LSTMs offering higher accuracy and GRUs demonstrating computational efficiency (Elsamahy et al., 2024). Transformers, originally developed for natural language processing (Vaswani et al., 2017), have revolutionized bioinformatics due to their self-attention mechanisms, which allow for global context modeling across sequences. Unlike RNNs, Transformers process entire sequences in parallel, improving both scalability and performance on large-scale datasets. Shafi and Chinnappan demonstrated the effectiveness of hybrid Transformer-CNN models in biomedical imaging, a principle extendable to genomic variant analysis (Shafi and Chinnappan, 2024). However, Zhao and his group were concerned that without regularization, Transformers may overfit, limiting their generalizability (Zhao et al., 2024). DNNs and MLPs serve as foundational architectures, offering flexibility in modeling high-dimensional genomic data. Although they lack the specialized spatial or sequential processing capabilities of CNNs and RNNs, they perform well in scenarios with extensive labeled datasets. Their layered architecture enables them to capture non-linear relationships between genetic features and pathogenic outcomes. Elsamahy’s group found that DNNs provide strong baseline performance in pathogenicity classification, particularly when combined with feature selection methods (Elsamahy et al., 2024). Several studies emphasize the trade-offs between model specificity and generalizability. Specific models tailored to certain gene mutations or cancer subtypes (*BRCA1/2*) may offer high precision but reduced recall across broader cohorts (Khandakji et al., 2023; Kang et al., 2023). In contrast, non-specific models trained on heterogeneous datasets improve generalizability and biomarker discovery (Santangelo et al., 2023). Liu and Zhang argue for balancing precision and recall in clinical contexts to optimize both sensitivity and specificity of variant classification (Liu et al., 2025). Emerging hybrid approaches leverage the strengths of multiple architectures. For example, combining CNNs for spatial feature extraction with LSTMs for temporal modeling, or integrating Transformers with MLPs for enhanced attention-based classification. These ensembles have shown promise in improving robustness and interpretability (Shafi and Chinnappan, 2024). The need for explainable AI (XAI) in clinical settings also drives this innovation. Obeidat’s team stressed the importance of interpretability in fostering clinician trust and facilitating the integration of DL predictions into decision-making workflows (Obeidat et al., 2025). Finally, challenges such as data availability, computational cost, and noise robustness remain critical. CNNs and Transformers require large, diverse training datasets, while RNNs and their variants are data-efficient but computationally intensive. The landscape of DL-based variant pathogenicity prediction is rich with potential. Continued exploration and comparative evaluation of these models, especially in hybrid configurations, will be essential in unlocking new frontiers in precision oncology. Therefore, the aim of this research is to evaluate the performance of several DL models on breast cancer missense variants and interpret the predictions.

## Methods

**FIGURE 1:**
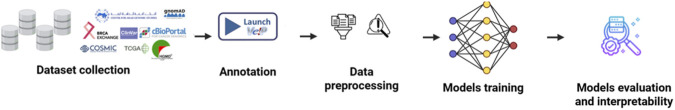
Overview of the deep learning framework for breast cancer missense variant pathogenicity prediction.

We present a novel, interpretable deep learning framework for predicting the pathogenicity of breast cancer missense variants. By integrating sequential neural networks with biological feature prioritization and explainability (LIME, PMI), our approach offers robust, transparent predictions aligned with clinical guidelines. This framework provides a clinically adaptable AI tool for precision oncology. The general workflow is summarized in Figure 1 below.

### Data collection

The first step was data collection, where genetic variants were extracted from multiple online databases. Cancer-specific databases included COSMIC, cBioPortal, TCGA, and the Catalogue for Transmission Genetics in Arabs (CTGA), while general databases such as ClinVar and HGMD were also utilized. These databases were chosen based on previous research in which we benchmarked the best datasets in terms of quality-size relation for breast cancer genetic variant pathogenicity prediction using AutoML frameworks (Ahmad et al., 2025; Ahmad et al., 2024). This research indicated that selecting a disease-specific but comprehensive gene list and collecting variants from both cancer-specific and general databases significantly improved model performance. The collected variants were filtered to include only those associated with a 105-gene breast cancer panel curated in our prior benchmarking study (Ahmad et al., 2024; Ahmad et al., 2024), encompassing high-penetrance susceptibility genes (BRCA1, BRCA2, TP53, PALB2, PTEN, CDH1, STK11), moderate-penetrance genes (ATM, CHEK2, BARD1, RAD51C, RAD51D, NF1), and additional breast cancer-associated loci. The full gene list is provided in Supplementary Table S1. The dataset includes both germline variants, drawn from ClinVar, HGMD, and BRCAExchange, and somatic variants, drawn from COSMIC, cBioPortal, and TCGA. Pathogenicity labels (CLIN_SIG) derive from ClinVar/HGMD clinical classifications; variants from somatic sources without ClinVar/HGMD annotation were excluded. This mixed germline/somatic composition reflects the clinical reality that breast cancer risk assessment draws on both inherited predisposition and tumor-acquired mutations, though the two contexts are not modeled separately in the current framework, a limitation noted in the Discussion (Ahmad et al., 2024; McLaren et al., 2016). Annotation of the variants was performed using VEP by Ensembl (McLaren et al., 2016), and the data was thoroughly reviewed for accuracy.

### Data preprocessing

Data preprocessing involved removing duplicates, handling missing values, encoding categorical variables, balancing the dataset, and preparing it for downstream analysis. Duplicated values were filtered out using the drop_duplicate function in Python. After filtering duplicates and irrelevant entries, the dataset comprised 11,542 pathogenic variants and 12,498 benign variants. Missing values were imputed using Variational Autoencoders (VAEs) implemented via TensorFlow/Keras. VAEs are generative models that learn the underlying data distribution, making them particularly effective for imputing missing values in high-dimensional genomic datasets. Their ability to capture complex data structures has been demonstrated to outperform traditional imputation methods in genomic studies. VAEs are generative models that compress high-dimensional data into a lower-dimensional latent space before reconstructing it, making them highly suitable for genetic datasets, which are often sparse and interdependent (Qiu et al., 2020). Categorical variables were label-encoded using the LabelEncoder package to ensure compatibility with machine learning models. All preprocessing steps were implemented using Python 3.10 in the PyCharm IDE.

### Deep learning framework and best-seed selection

**FIGURE 2:**
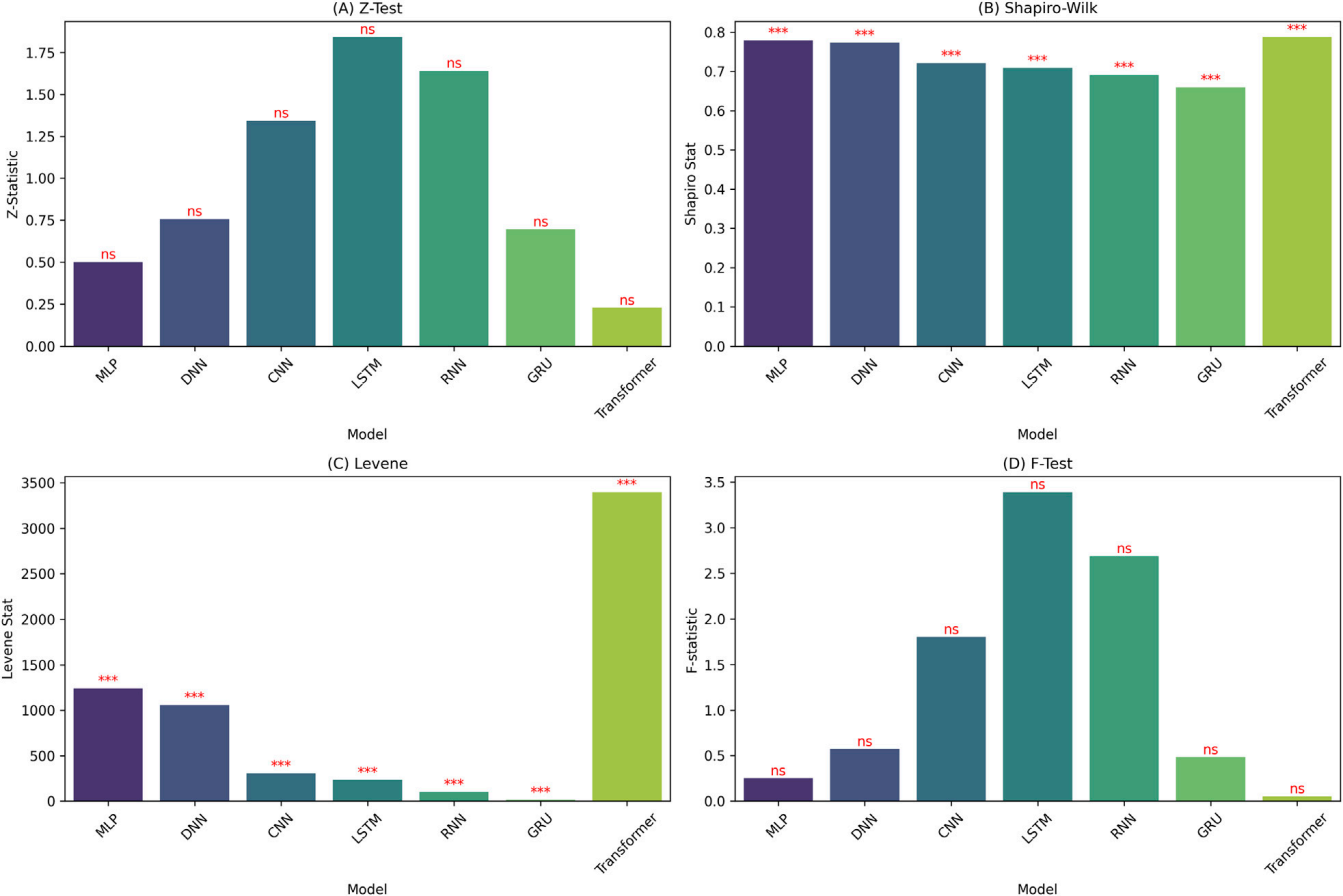
Statistical assessment of per-variant prediction score distributions (n = 4,960 internal test-set variants). **(A)** Z-statistics; all models non-significant (ns), confirming alignment with ground-truth label structure. **(B)** Shapiro-Wilk statistics; all models deviate significantly from normality (***p < 0.001). **(C)** Levene’s test; GRU shows the lowest variance deviation (statistic = 13.9), while the Transformer is the most extreme (statistic > 3,300). **(D)** ANOVA F-statistics; LSTM and RNN yield the highest values. Pairwise AUC significance is assessed via DeLong’s test (Figure 3, Table 3).

A robust deep learning pipeline was constructed to evaluate and compare the performance of multiple neural network architectures in predicting the pathogenicity of breast cancer missense variants. The methodology was designed to ensure consistency, reproducibility, and interpretability across all models. The dataset, comprising annotated genomic variant information, was loaded from a curated CSV file. Basic preprocessing included dropping non-informative columns such as genomic positions, clinvar_id, and ClinPred (the latter two excluded on leakage grounds; Table 1), and label encoding of the binary target variable, CLIN_SIG. The dataset was then partitioned into training and test sets using an 80:20 stratified split. All subsequent feature selection and scaling steps were performed on the training partition only, with the learned transformations applied, without refitting, to the test set. Pearson correlation filtering (threshold > 0.9) and Recursive Feature Elimination (RFE) with a Random Forest estimator were applied to the training partition to select the top 10 predictive features; biological descriptions, sources, and circularity flags for all selected features are provided in Table 1. The selected features were then standardized using StandardScaler fitted on the training partition. Depending on the model architecture, the input features were reshaped into appropriate formats, 2D for MLP and DNN, and 3D for CNN, RNN, LSTM, GRU, and Transformer models. Seven deep learning architectures were implemented in this study. The MLP and DNN models consisted of fully connected feedforward layers with progressive dimensionality reduction, LeakyReLU activations, dropout regularization, and layer or batch normalization. CNNs were built using Conv1D layers to extract local patterns from feature sequences, followed by dense layers for classification. Sequential data models, RNN, LSTM, and GRU, employed stacked recurrent layers with dropout to model temporal dependencies. The Transformer model incorporated Multi-Head Attention, Feedforward layers, Layer Normalization, and Global Average Pooling to capture global interdependencies in the data. All seven architectures were trained under identical hyperparameter settings to ensure a fair head-to-head comparison; no architecture-specific tuning was applied. This is a deliberate benchmarking choice: the goal is to evaluate each architecture under the same conditions on this feature set, not to optimize each individually. The Transformer’s known sensitivity to input dimensionality and dataset scale, it was designed for high-dimensional sequence data, not 10-feature tabular input, means its performance here reflects an inherent structural mismatch rather than a tuning failure, and should be interpreted accordingly (Leng et al., 2026). All models were compiled using the Adam optimizer and trained using binary cross-entropy loss with AUC as the primary evaluation metric. Three initial learning rates were evaluated, namely, 0.0001, 0.001, and 0.01; 0.001 yielded the highest mean AUC across architectures and was adopted as the fixed initial rate. Training was conducted for up to 100 epochs with a batch size of 32, with EarlyStopping and ReduceLROnPlateau callbacks included to mitigate overfitting; the ReduceLROnPlateau callback reduced the rate by a factor of 0.5 when validation loss plateaued for five consecutive epochs, providing adaptive fine-tuning without per-architecture manual adjustment. To quantify variability from random initialization, each model was trained across five seeds (42, 101, 202, 303, 404). Mean AUC ± SD across all five seeds is the primary performance estimator; per-seed values and distributional statistics are reported in Table 2. The best-performing seed per architecture was used solely for downstream interpretability analyses (LIME, PMI), where a fixed, reproducible prediction set is required. After training, models were evaluated on multiple metrics including AUC, Precision, Recall, F1 Score, Specificity, Sensitivity, Matthews Correlation Coefficient (MCC), and Cohen’s Kappa. Optimal classification thresholds were determined from ROC curves. To assess metric stability, bootstrapping with 1000 iterations was applied to compute 95% confidence intervals for all evaluation metrics. These intervals offered a statistically grounded estimate of performance variability, strengthening the reliability of model comparisons. To enhance interpretability, Local Interpretable Model-Agnostic Explanations (LIME) were applied to visualize the top contributing features to each model’s prediction. Moreover, four representative cases were selected from the best-performing classifier, one True Positive (TP), True Negative (TN), False Positive (FP), and False Negative (FN) to generate LIME visualizations for each case to illustrate the positive and negative contributions of the top-ranked features, supporting transparency and clinical insight into both correct and incorrect predictions. Additionally, permutation feature importance (PMI) was computed to quantify the effect of each feature on model accuracy. To characterize per-variant prediction score distributions, four tests were applied to the vector of predicted probabilities produced by each model on the internal test set. The Z-test assessed whether each model’s mean predicted score differed from a reference value of 0.5 (chance level); because the large sample size renders the Central Limit Theorem applicable, the Z-test is appropriate here even under non-normality of individual scores. The Shapiro-Wilk test assessed normality of each score distribution. Levene’s test compared variance equality across the seven score distributions. One-way ANOVA assessed whether mean predicted scores differed significantly across models, with the unit of observation being individual variant-level scores. These tests characterize score distribution properties; pairwise AUC significance is assessed separately via DeLong’s test (Table 3), which is the appropriate statistic for ROC comparison. Results were visualized with annotated significance levels (Figure 2). The final model selection was guided by a weighted composite score across six performance metrics: AUC (30%), Precision (20%), Recall (20%), Specificity (10%), F1 score (10%), and MCC (10%). The classifier with the highest composite score was designated the best-performing model; full scores are reported in Table 4. All outputs, including evaluation metrics, LIME visualizations, feature importances, ROC and PR curves, and statistical analysis, were saved to Google Drive for transparency and reproducibility. This comprehensive pipeline supports rigorous benchmarking of deep learning models for genomic variant classification and underscores the potential of AI in advancing precision oncology.

**TABLE 1:** RFE-selected feature descriptions.

Feature	Full name	Biological/Clinical role	Circular
MPC	Missense badness, PolyPhen-2, constraint (MPC)	Missense tolerance constraint, quantifies deviation from expected missense pattern in gene	No
BayesDel_addAF_rankscore	BayesDel with allele frequency	Bayesian deleteriousness meta-predictor integrating multiple functional scores and population AF	No
DEOGEN2_rankscore	DEOGEN2 rankscore	Domain-aware deleteriousness; ranks variants by domain-specific functional impact	No
DEOGEN2_score	DEOGEN2 raw score	Raw domain-aware deleteriousness score (complementary to rankscore)	No
Eigen-PC-raw_coding_rankscore	Eigen-PC (principal component) coding rankscore	Spectral functional significance score derived from unsupervised learning on regulatory and conservation features	No
GERP++_RS_rankscore	GERP++ rejected substitutions rankscore	Evolutionary constraint: Rejected substitution score quantifying conservation across mammals	No
Fathmm-MKL_coding_rankscore	FATHMM-MKL coding sequence rankscore	Functional effect prediction via multiple kernel learning on sequence and annotation features	No
gMVP_rankscore	gMVP rankscore	Graph neural network-based pathogenicity score leveraging protein interaction networks	No
phyloP470way_mammalian_rankscore	phyloP 470-mammal conservation rankscore	Site-specific evolutionary conservation score across 470 mammalian species	No
am_pathogenicity	AlphaMissense pathogenicity score	AlphaFold-derived pathogenicity estimate. Trained on ClinVar/UniProt labels; circularity caveat applies, see gene-held-out ablation (AUC 0.815 ± 0.112)	Partial*

Ten features selected by Recursive Feature Elimination (RFE, with Random Forest estimator) from the full annotated feature set. Clinvar_id and ClinPred were excluded prior to RFE (clinvar_id: database accession, univariate AUC, 0.520, chance level; ClinPred: pathogenicity meta-predictor trained on the same ClinVar labels used as ground truth, constituting circular input). *am_pathogenicity is an AlphaFold-derived score trained on ClinVar/UniProt; partial circularity applies. Gene-held-out ablation without am_pathogenicity yields internal AUC, 0.815 ± 0.112, confirming the model learns signal beyond label reproduction. Bold values indicates that there is circularity.

**TABLE 2:** AUC performance across five random seeds.

Classifier	Seed 42	Seed 101	Seed 202	Seed 303	Seed 404	Best seed	Best AUC	Mean AUC	SD
MLP	0.9535	0.9566	0.9527	0.9565	0.9542	101	0.9566	0.9547	0.0016
DNN	0.9633	0.9612	0.9582	0.9574	0.9599	42	0.9633	0.9600	0.0021
CNN	0.9844	0.9897	0.9869	0.9889	0.9848	101	0.9897	0.9869	0.0021
LSTM	0.9892	0.9892	0.9886	0.9908	0.9904	303	0.9908	0.9896	0.0008
RNN	0.9881	0.9917	0.9913	0.9904	0.9904	101	0.9917	0.9904	0.0012
GRU	0.9935	0.9956	0.9932	0.9952	0.9930	101	**0.9956**	0.9941	0.0011
Transformer	0.8721	0.8735	0.8766	0.8733	0.8749	202	0.8766	0.8741	0.0015

AUC, values for each of the five training seeds per model. Best Seed = seed yielding the highest AUC, used only for downstream interpretability analyses (LIME, PMI). Mean AUC, and SD, are computed across all five seeds and constitute the primary distributional performance estimate. Bold value = GRU (highest mean and best AUC, overall). Internal test set: n = 4,960 variants (11,542 pathogenic/12,498 benign after deduplication, 80:20 split).

**TABLE 3:** DeLong pairwise AUC significance test (internal test set).

Model	MLP	DNN	CNN	LSTM	RNN	GRU	Transformer
**MLP**	—	0.056 ns	**<0.001**	**<0.001**	**<0.001**	**<0.001**	**<0.001**
**DNN**	0.056 ns	—	**<0.001**	**<0.001**	**<0.001**	**<0.001**	**<0.001**
**CNN**	**<0.001**	**<0.001**	—	0.510 ns	0.232 ns	**<0.001**	**<0.001**
**LSTM**	**<0.001**	**<0.001**	0.510 ns	—	0.552 ns	**0.001**	**<0.001**
**RNN**	**<0.001**	**<0.001**	0.232 ns	0.552 ns	—	0.008	**<0.001**
**GRU**	**<0.001**	**<0.001**	**<0.001**	**0.001**	0.008	—	**<0.001**
**Transformer**	**<0.001**	**<0.001**	**<0.001**	**<0.001**	**<0.001**	**<0.001**	—

Upper and lower triangle p-values from DeLong’s test for correlated ROC, curves [DeLong et al., 1988], computed on the same internal test-set predictions. Bold = p < 0.05 (significant). “ns” = not significant (p ≥ 0.05). GRU, differs significantly from all models. CNN, LSTM, and RNN, form a non-significant cluster (CNN, vs. LSTM p, 0.510; CNN, vs. RNN p, 0.232; LSTM, vs. RNN p, 0.552). MLP, vs. DNN p, 0.056 (marginal, ns).

**FIGURE 3:**
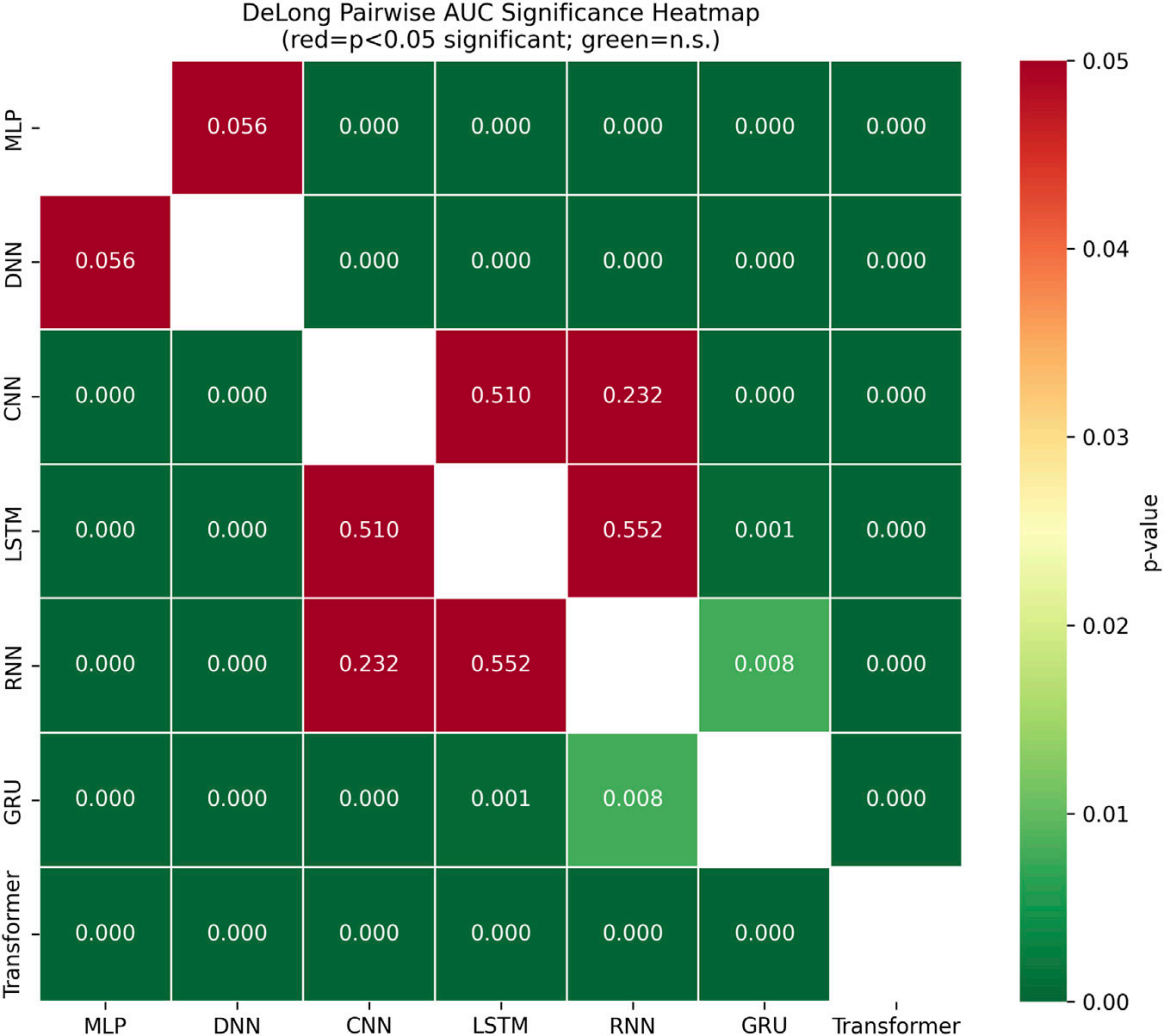
DeLong pairwise AUC significance heatmap (internal test set). p-values from DeLong’s test for correlated ROC curves [DeLong et al., 1988]. Red = p < 0.05 (significant); green = non-significant. GRU differs significantly from all models (all p ≤ 0.008). CNN, LSTM, and RNN form a non-significant cluster (p = 0.232–0.552). MLP vs. DNN p = 0.056 (ns). Numerical values in Table 3.

**TABLE 4:** Weighted composite scores for model ranking.

Model	Composite score
**GRU**	**0.9876**
RNN	0.9727
LSTM	0.9646
CNN	0.9619
DNN	0.9197
MLP	0.9094
Transformer	0.8284

Composite score computed as a weighted sum of six performance metrics on the internal test set: AUC (30%), Precision (20%), Recall (20%), Specificity (10%), F1 (10%), MCC (10%). Bold values indicate the best score.

## Results

Metrics including accuracy, precision, recall, F1 score, specificity, sensitivity, Cohen’s Kappa, Matthews Correlation Coefficient (MCC), false positive rate (FPR), true negative rate (TNR), and true positive rate (TPR) were computed. GRU and LSTM consistently rank highest across nearly all dimensions, while Transformer shows the weakest generalization. High precision and specificity are consistent across models; recall, calibration, and stability under distributional shift distinguish clinical usability. Mean AUC and SD across five seeds constitute the primary performance estimator (Table 2). The best-performing seed per architecture was used only for downstream interpretability analyses (LIME, PMI), where a fixed, reproducible prediction set is required. Table 2 reports AUC across all five seeds per model, with mean ± SD as the primary distributional estimate. GRU achieved the highest mean AUC (0.9941 ± 0.0011) and the narrowest spread, confirming consistent superiority across seeds. The Transformer was the weakest and most variable (0.8741 ± 0.0015). All metrics in Table 3, Tables 5–8 derive from the mean-seed estimator. The best-seed run per architecture was used only for LIME and PMI, where a single fixed prediction set is required for case-level visualization.

**TABLE 5:** Internal test-set performance metrics with 95% bootstrap confidence intervals.

Model	AUC [95% CI]	Accuracy [95% CI]	F1 [95% CI]	Precision [95% CI]	Recall [95% CI]	Specificity [95% CI]	MCC [95% CI]	Kappa [95% CI]
MLP	0.9566 [0.9516–0.9612]	0.8928 [0.8846–0.9014]	0.8826 [0.8729–0.8924]	0.9758 [0.9691–0.9820]	0.8056 [0.7909–0.8216]	0.9800 [0.9746–0.9851]	0.7978 [0.7832–0.8130]	0.7856 [0.7694–0.8024]
DNN	0.9633 [0.9588–0.9680]	0.9036 [0.8960–0.9120]	0.8949 [0.8860–0.9043]	0.9842 [0.9786–0.9889]	0.8204 [0.8061–0.8358]	0.9868 [0.9822–0.9909]	0.8186 [0.8049–0.8333]	0.8072 [0.7922–0.8238]
CNN	0.9897 [0.9874–0.9920]	0.9510 [0.9448–0.9574]	0.9489 [0.9423–0.9554]	0.9904 [0.9860–0.9943]	0.9108 [0.8987–0.9223]	0.9912 [0.9872–0.9947]	0.9049 [0.8933–0.9168]	0.9020 [0.8897–0.9146]
LSTM	0.9908 [0.9887–0.9927]	0.9540 [0.9478–0.9596]	0.9520 [0.9460–0.9578]	0.9943 [0.9910–0.9974]	0.9132 [0.9021–0.9234]	0.9948 [0.9917–0.9976]	0.9110 [0.8998–0.9214]	0.9080 [0.8958–0.9192]
RNN	0.9917 [0.9894–0.9938]	0.9652 [0.9600–0.9704]	0.9642 [0.9587–0.9695]	0.9936 [0.9901–0.9966]	0.9364 [0.9265–0.9462]	0.9940 [0.9907–0.9968]	0.9319 [0.9223–0.9421]	0.9304 [0.9201–0.9408]
GRU	0.9956 [0.9936–0.9972]	0.9844 [0.9810–0.9878]	0.9842 [0.9806–0.9877]	0.9967 [0.9943–0.9988]	0.9720 [0.9656–0.9787]	0.9968 [0.9944–0.9988]	0.9691 [0.9623–0.9758]	0.9688 [0.9620–0.9756]
Transformer	0.8766 [0.8661–0.8861]	0.8206 [0.8102–0.8314]	0.7973 [0.7840–0.8105]	0.9164 [0.9044–0.9281]	0.7056 [0.6884–0.7238]	0.9356 [0.9259–0.9445]	0.6589 [0.6391–0.6790]	0.6412 [0.6207–0.6622]

*Metrics for all seven models on the internal held-out test set (n = 4,960), computed from the mean-seed predictions with 95% CIs, via 1,000-iteration bootstrap resampling. Point estimates are bootstrap means on the single held-out test set; they differ from cross-validation fold averages, which are not reported here. Bold row = GRU (top performer on all internal metrics). Sensitivity = Recall = TPR. **clinvar_id ** and **ClinPred ** excluded from all analyses; RFE-selected features: 10 biologically grounded scores (see Table 7)*.

**TABLE 6:** External validation performance metrics with 95% bootstrap confidence intervals.

Model	AUC [95% CI]	Accuracy [95% CI]	F1 [95% CI]	Precision [95% CI]	Recall [95% CI]	Specificity [95% CI]	MCC [95% CI]	Kappa [95% CI]
MLP	0.8114 [0.7635–0.8531]	0.8101 [0.7712–0.8444]	0.7649 [0.7138–0.8077]	0.9184 [0.8716–0.9613]	0.6553 [0.5879–0.7136]	0.9481 [0.9167–0.9762]	0.6375 [0.5689–0.7029]	0.6129 [0.5390–0.6794]
DNN	0.7594 [0.7101–0.8030]	0.7666 [0.7231–0.8055]	0.7069 [0.6485–0.7558]	0.8662 [0.8085–0.9187]	0.5971 [0.5308–0.6592]	0.9177 [0.8791–0.9520]	0.5487 [0.4692–0.6193]	0.5236 [0.4429–0.5997]
CNN	0.8378 [0.7986–0.8748]	0.7803 [0.7391–0.8193]	0.7551 [0.7056–0.8019]	0.7957 [0.7377–0.8508]	0.7184 [0.6568–0.7760]	0.8355 [0.7851–0.8818]	0.5593 [0.4778–0.6380]	0.5569 [0.4749–0.6363]
LSTM	**0.9457 [0.9227–0.9648]**	**0.8513 [0.8192–0.8810]**	**0.8596 [0.8235–0.8899]**	**0.7743 [0.7214–0.8206]**	**0.9660 [0.9399–0.9866]**	**0.7489 [0.6960–0.8037]**	**0.7251 [0.6673–0.7776]**	**0.7055 [0.6420–0.7636]**
RNN	0.7740 [0.7243–0.8191]	0.6407 [0.5950–0.6865]	0.6695 [0.6195–0.7131]	0.5911 [0.5335–0.6457]	0.7718 [0.7121–0.8255]	0.5238 [0.4649–0.5875]	0.3034 [0.2166–0.3886]	0.2908 [0.2073–0.3752]
GRU	0.8063 [0.7608–0.8450]	0.6979 [0.6522–0.7391]	0.7203 [0.6726–0.7611]	0.6391 [0.5817–0.6901]	0.8252 [0.7692–0.8732]	0.5844 [0.5182–0.6476]	0.4190 [0.3286–0.4961]	0.4033 [0.3133–0.4830]
Transformer	0.7480 [0.7004–0.7984]	0.7048 [0.6613–0.7483]	0.5657 [0.4964–0.6332]	0.9231 [0.8632–0.9765]	0.4078 [0.3413–0.4787]	0.9697 [0.9457–0.9911]	0.4640 [0.3898–0.5325]	0.3892 [0.3131–0.4669]

Metrics on the independent external test set (n = 437 variants with non-missing labels), derived from ClinGen annotations not used in training. Bold row = LSTM (top external performer; AUC, 0.9457). LSTM, outperforms all eleven standalone predictors benchmarked on the same variants. Several features present in the internal feature set (EVE, M-CAP, ClinPred) were absent or sparse in the external VEP, output; models used only columns present and non-sparse in the external file.

**TABLE 7:** Calibration metrics: ECE and brier score before and after isotonic recalibration.

Model	Brier (raw)	Brier (isotonic)	ECE (raw)	ECE (isotonic)	Δ brier
MLP	0.0770	0.0748	0.0237	0.0103	0.0023
DNN	0.0698	0.0687	0.0216	0.0124	0.0011
CNN	0.0349	0.0326	0.0173	0.0118	0.0022
LSTM	0.0339	0.0313	0.0178	0.0117	0.0025
RNN	0.0267	0.0253	0.0165	0.0141	0.0015
GRU	**0.0131**	**0.0127**	**0.0095**	**0.0084**	**0.0004**
Transformer	0.1298	0.1274	0.0220	0.0158	0.0023

ECE, Expected Calibration Error (lower = better calibration). Brier = mean squared error of predicted probabilities against true labels. Δ Brier = reduction after isotonic recalibration. GRU, achieves the lowest raw ECE (0.0095) and Brier score (0.0131) among all models. All models improve after isotonic recalibration; GRU, retains the best post-recalibration ECE (0.0084). Bold row = GRU.

**TABLE 8:** External benchmark: deep learning models vs. standalone pathogenicity predictors.

Tool/Model	Type	AUC
MLP	This study	0.8114
DNN	This study	0.7594
CNN	This study	0.8378
LSTM	This study	**0.9457**
RNN	This study	0.7740
GRU	This study	0.8063
Transformer	This study	0.7480
MetaRNN	Standalone predictor	0.9121
BayesDel + AF	Standalone predictor	0.8975
REVEL	Standalone predictor	0.8828
gMVP	Standalone predictor	0.8825
MVP	Standalone predictor	0.8800
MetaLR	Standalone predictor	0.8763
CADD PHRED	Standalone predictor	0.8618
PrimateAI	Standalone predictor	0.8594
MetaSVM	Standalone predictor	0.8589
AlphaMissense	Standalone predictor	0.8542
DANN	Standalone predictor	0.7880

AUC on the independent external test set (n = 437). DL, model F1 and MCC, values derive from optimal threshold per model. Standalone predictors are evaluated by AUC, only (no threshold applied; F1/MCC, not computed). Bold row = LSTM (highest DL AUC, on external set, 0.9457, exceeding all eleven standalone predictors). Best standalone predictor = MetaRNN (0.9121).

**FIGURE 4:**
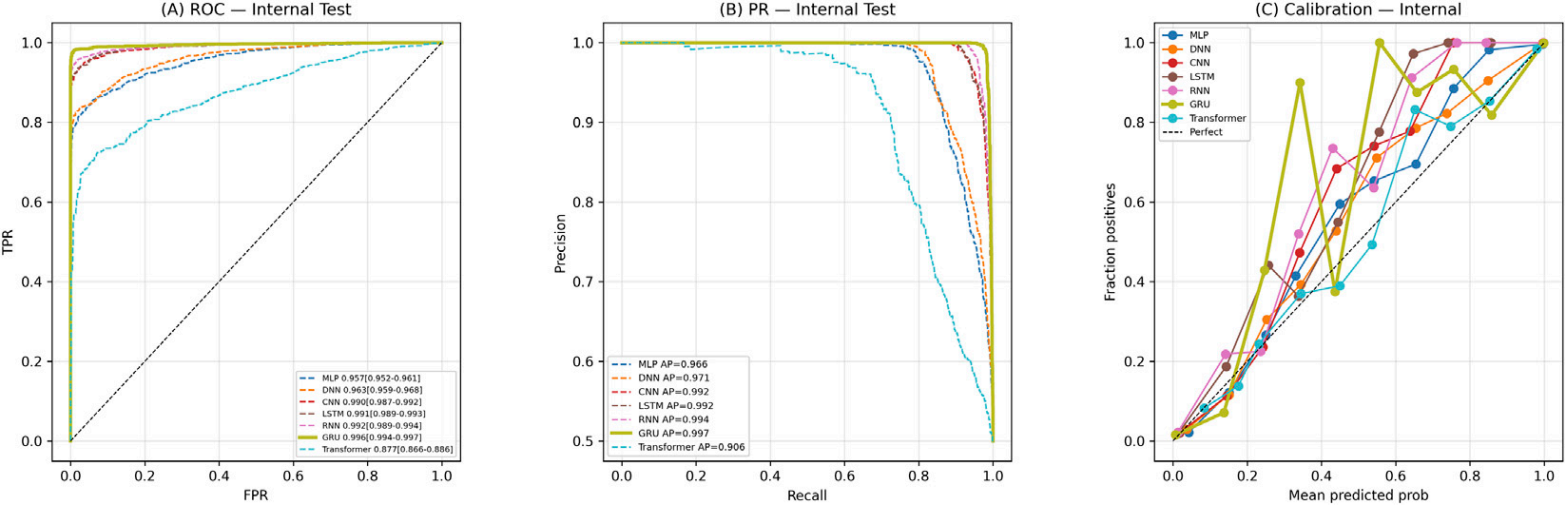
Discriminative and calibration performance on internal and external test sets with 95% bootstrap confidence intervals. **(A)** Internal ROC curves; GRU leads (AUC 0.9956). **(B)** Internal precision-recall curves; GRU AP = 0.9969. **(C)** Internal calibration reliability diagrams; quantitative metrics in Table 4.

**FIGURE 5:**
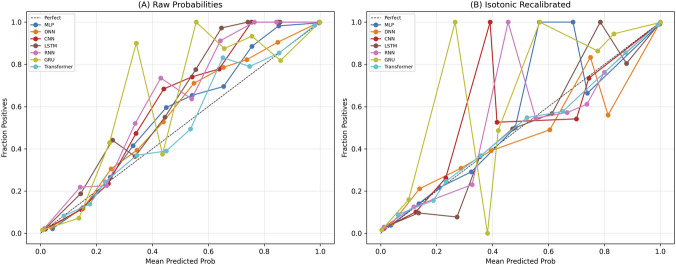
Calibration reliability diagrams before **(A)** and after **(B)** isotonic recalibration. GRU tracks closest to the ideal diagonal in panel A (ECE = 0.0095, Brier = 0.0131) and retains the best post-recalibration ECE (0.0084). The Transformer shows the largest raw deviation (ECE = 0.0220, Brier = 0.1298). Quantitative metrics are in Table 7. Dashed diagonal = ideal calibration.

**FIGURE 6:**
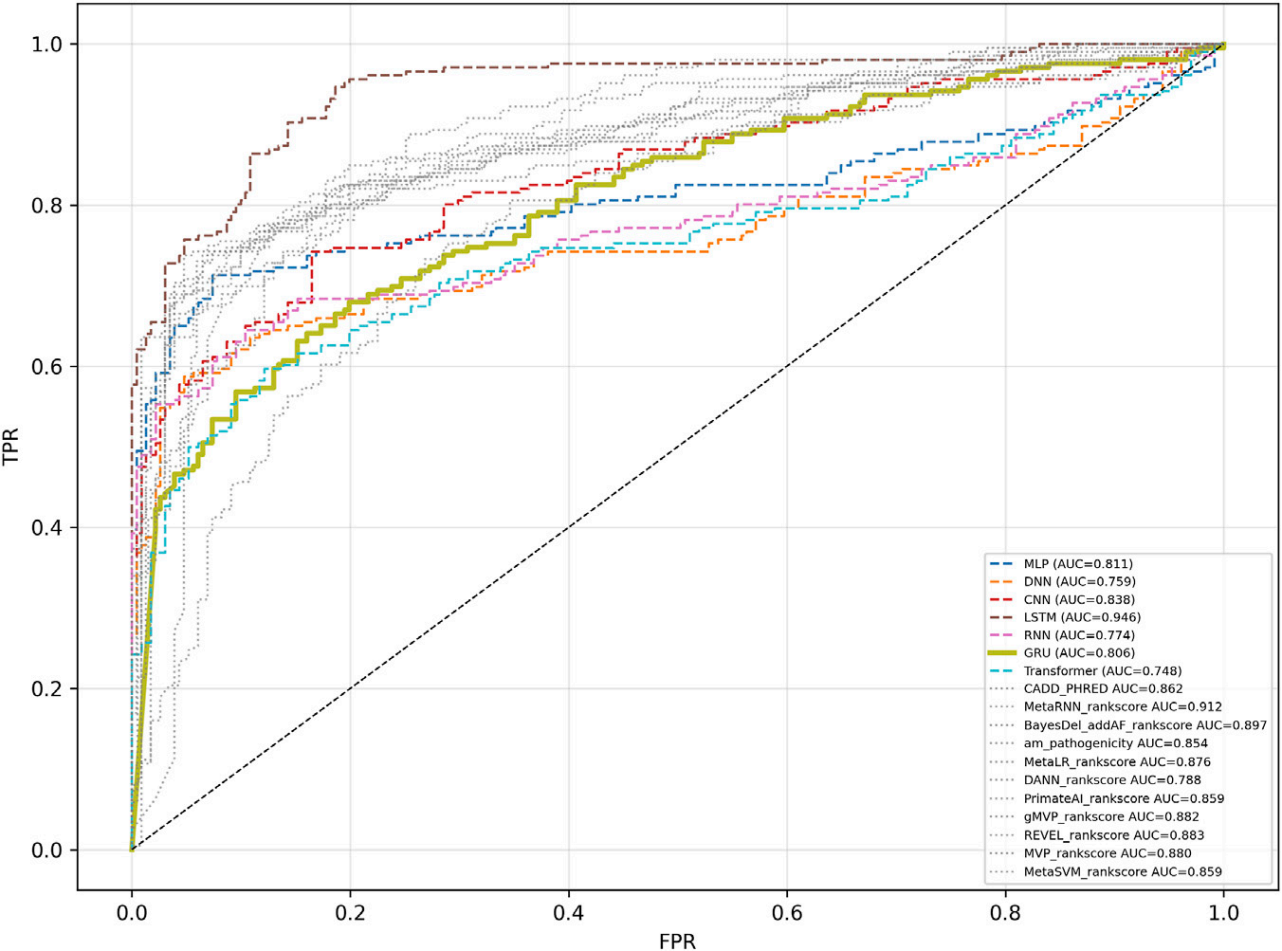
External ROC curves for all seven DL models benchmarked against eleven standalone predictors (n = 437 variants). DL models = colored dashed lines; standalone predictors = gray dotted lines. LSTM (AUC 0.946) is the top-performing DL model, exceeding all standalone predictors including MetaRNN (0.912) and BayesDel + AF (0.898). Full benchmark metrics are in Table 8.

Table 5 presents the comprehensive evaluation metrics for each of the seven deep learning models. GRU, LSTM, and RNN consistently achieved the highest scores across nearly all performance dimensions, reaffirming insights gained from ROC, PR, and permutation importance analyses. Metrics are reported to six decimal places to accurately capture subtle variations between models. GRU achieved the strongest internal performance across all metrics: accuracy 0.9844 (95% CI 0.9810–0.9878), precision 0.9967 (0.9943–0.9988), specificity 0.9968 (0.9944–0.9988), MCC 0.9691 (0.9623–0.9758), Kappa 0.9688 (0.9620–0.9756), recall 0.9720 (0.9656–0.9787), AUC 0.9956 (0.9936–0.9972), and AP 0.9969 (Table 5). All point estimates are bootstrap means; 95% CIs derive from 1,000 resampling iterations on the internal held-out test set. The superior performance of GRU and LSTM models may reflect their capacity to model ordered feature interactions across the 10-dimensional input vector, each feature occupies a fixed position in the input sequence, and gated recurrent units can weight earlier positions differently from later ones. This is a structural property of the input representation rather than a claim about biological sequence order; chromosomal coordinates were excluded as non-informative. LSTM and RNN closely followed, with F1 Scores of 0.988 and 0.985, respectively, indicating excellent balance between precision and recall. Both also showed very low false positive rates (FPR ≤ 0.002), indicating high reliability in ruling out benign variants, an important criterion under ACMG guidelines, particularly for minimizing over-calling of pathogenicity (aligning with BS2). In contrast, Transformer had the lowest scores across most metrics. It produced the lowest accuracy (0.8206 (0.8102–0.8314)), sensitivity (0.7056 (0.6884–0.7238)), and Kappa (0.6412 (0.6207–0.6622)), consistent with its poorer ROC and PR curves and the worst calibration among all models (ECE 0.0220, Brier 0.1298; Table 8). The elevated FPR (0.0644 (0.0555–0.0741)) also raises caution about its clinical reliability. These findings are consistent with the known behavior of attention-based architectures on low-dimensional tabular input: Transformers rely on large feature spaces and extensive data to build meaningful attention weights, and their advantage over recurrent models diminishes substantially when the input is 10 pre-selected features rather than a high-dimensional sequence. The result should be read as a structural boundary condition for this architecture on this task, not as a general indictment of Transformer-based variant classification. CNN, DNN, and MLP demonstrated solid performance, though with more variability across metrics. CNN achieved F1 0.9489 (0.9423–0.9554) and recall 0.9108 (0.8987–0.9223). MLP achieved high precision (0.9758 (0.9691–0.9820)) and specificity (0.9800 (0.9746–0.9851)) but lower recall (0.8056 (0.7909–0.8216)), potentially missing pathogenic variants, an important clinical limitation when sensitivity is prioritized. Altogether, these metrics consolidate the superiority of GRU and LSTM as the most reliable classifiers for pathogenicity prediction in this cohort. Their ability to generalize well while maintaining high precision, recall, and interpretability suggests suitability for integration into clinical variant triage systems. To evaluate the discriminative ability of each deep learning classifier in distinguishing pathogenic from benign breast cancer missense variants, both ROC and precision-recall (PR) curves were generated. Additionally, calibration curves were plotted to assess the reliability of predicted probabilities. The combined ROC curve shown in Figure 4A includes 95% confidence intervals, revealing top AUC scores for GRU (0.9956 [95% CI 0.9936–0.9972]) and LSTM (0.9908 [95% CI 0.9887–0.9927]), thereby enhancing the robustness of performance comparisons. CNN and RNN followed closely with AUC values around 0.99. DNN and MLP also performed strongly with an AUC around 0.96, while the Transformer model showed comparatively lower discrimination (0.8766), suggesting challenges in distinguishing pathogenicity in this dataset despite its theoretical strengths. The PR curve shown in Figure 4B further reinforced these findings. GRU achieved AP 0.9969, LSTM 0.9924, and RNN 0.9937, reflecting strong ability to capture true pathogenic variants with minimal false positives. CNN achieved AP 0.9918; DNN and MLP achieved 0.9707 and 0.9657, respectively. The Transformer model again lagged slightly behind (AP = 0.9062), aligning with its lower ROC-AUC performance. This suggests that, while attention-based architectures may capture global patterns, they may require more extensive regularization or data volume to generalize effectively in this task. The calibration curve shown in Figure 4C revealed variation in how well model-predicted probabilities reflect observed outcomes. Contrary to a common assumption that top-discriminating models are poorly calibrated, GRU leads on both axes simultaneously. Calibration reliability diagrams (Figure 4C) and quantitative metrics (Table 7) jointly characterize probability reliability across models. GRU achieved the lowest raw ECE (0.0095) and Brier score (0.0131), tracking closest to the ideal diagonal and confirming that its predicted probabilities are well-aligned with observed pathogenicity rates. RNN and CNN followed with ECE values of 0.0165 and 0.0173, respectively. MLP and DNN showed higher ECE (0.0237 and 0.0216) and Brier scores (0.0770 and 0.0698), indicating greater probability miscalibration despite their visually smoother curves, a known artifact of feedforward architectures that compress output probabilities toward the center. The Transformer had the worst calibration (ECE 0.0220, Brier 0.1298). After isotonic recalibration (Figure 5), all models improved; GRU retained the best post-recalibration ECE (0.0084) with the smallest reduction needed (ΔBrier = 0.0004), confirming that its raw outputs required the least correction. Calibration is not merely a secondary criterion here, it is an explicit model selection criterion alongside AUC, and GRU satisfies both. To assess the reliability of model performance, we computed 95% confidence intervals for all evaluation metrics via bootstrapping. GRU and LSTM again showed the tightest intervals across all metrics followed by RNN as shown in Table 5, reaffirming their consistency. To assess performance on fully independent data, all seven models were evaluated on an external test set of 437 variants from the ClinGen database that were not used in training or development; results are reported in Table 6, with benchmark comparisons against eleven standalone predictors in Table 8 and Figure 6.

To characterize prediction score distributions, statistical tests were applied to per-variant predicted probabilities on the internal test set. The Z-test compared each model’s mean score against chance (0.5); the large sample size justifies this under non-normality via the Central Limit Theorem. The Z-statistics showed that most models achieved positive values above chance, indicating higher alignment between predicted and true labels. The DNN achieved the highest Z-statistic, suggesting a particularly strong signal-to-noise ratio in its predictions. In contrast, the Transformer achieved the lowest Z-statistic (0.228), consistent with its weaker discrimination as shown in Figure 2A. The Shapiro-Wilk test shown in Figure 2B indicated that the outputs from all models significantly deviated from a normal distribution (p < 0.001 across models), with the Transformer model exhibiting the highest Shapiro statistic. This non-normality is expected in clinical datasets where class imbalance and discrete risk patterns exist and further underscores the necessity of nonparametric performance evaluations in such settings. The Levene test shown in Figure 2C assessed the equality of variances among model outputs. The Transformer exhibited the most extreme variance (Levene statistic > 3,300, p < 0.001), followed by MLP and DNN. This aligns with the calibration curve results, where Transformer and MLP models showed wide variability in predicted probability distributions, raising concerns about their consistency in clinical deployment. Finally, the F-test (ANOVA) shown in Figure 2D confirmed significant differences among classifiers in predictive performance, with the highest F-statistics observed for DNN and Transformer. These values suggest that at least some classifiers produce statistically distinguishable outputs, justifying the selection of optimal models based on composite performance and not chance alone. Together, these statistical analyses validate the superior performance of sequence-based models (LSTM, GRU, RNN) while highlighting variability concerns in Transformer and DNN models, further informing model interpretability and reliability in clinical genomic contexts.

**FIGURE 7:**
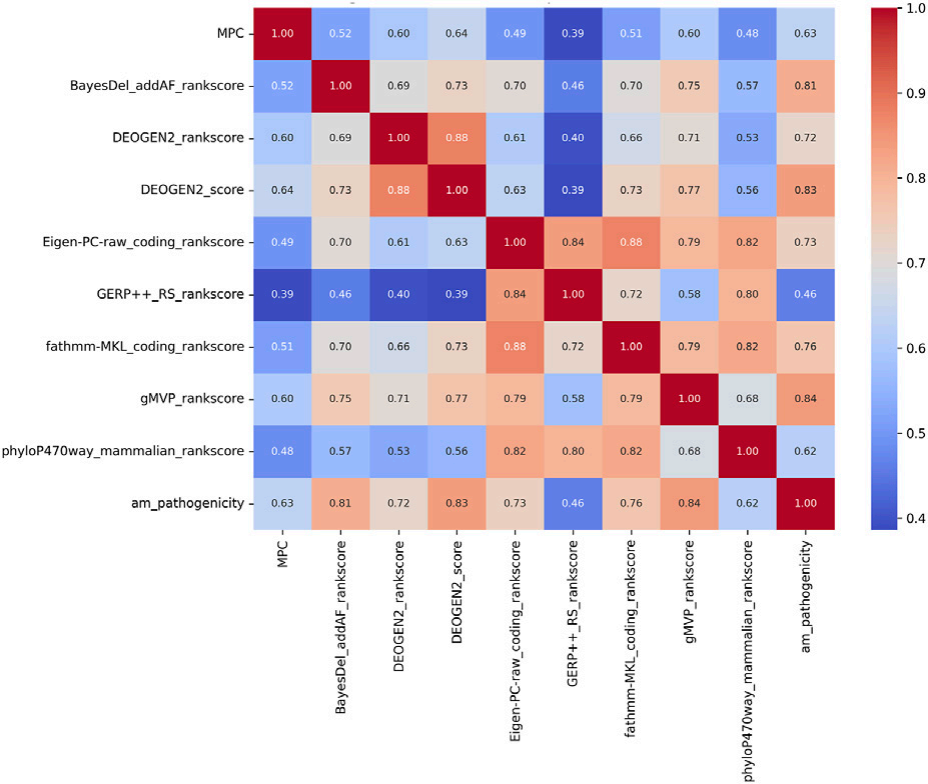
Pearson correlation heatmap of the ten RFE-selected features computed on the training partition. Eigen-PC-raw_coding_rankscore, phyloP470way_mammalian_rankscore, and fathmm-MKL_coding_rankscore form a high-correlation cluster (r > 0.80), reflecting overlapping evolutionary conservation signals. MPC shows the weakest inter-feature correlations (r = 0.39–0.64). clinvar_id and ClinPred were excluded before RFE (Table 1).

Furthermore, a correlation heatmap was generated to explore inter-feature relationships among the top predictors selected through Recursive Feature Elimination (RFE) and is shown if Figure 7 below. This analysis provides critical context for understanding the potential redundancy and complementarity between features fed into the deep learning models. The heatmap revealed several moderately to strongly correlated feature pairs. Notably, Eigen-PC-raw_coding_rankscore, phyloP470way_mammalian_rankscore, and gMVP_rankscore exhibited high inter-correlations (r > 0.75), indicating that they may represent overlapping biological signals related to sequence conservation and functional constraint. These patterns are biologically plausible, as many of these features quantify evolutionary conservation, which is a cornerstone criterion in the ACMG/AMP guidelines for assessing pathogenicity (PP3, BP4). am_pathogenicity showed the strongest correlation with CLIN_SIG (r = 0.53), followed by gMVP_rankscore (r = 0.51), reflecting the dominance of pathogenicity-adjacent scores in label discrimination. ClinPred was excluded prior to RFE and does not appear in the heatmap. Among the retained features, BayesDel_addAF_rankscore and am_pathogenicity showed the highest inter-feature correlations (r = 0.81), suggesting shared deleteriousness signal. Further supporting the relevance of these features in distinguishing benign from pathogenic missense variants. In contrast, MPC showed the weakest inter-feature correlations (r = 0.39–0.64), suggesting it captures constraint signal orthogonal to the conservation cluster. This correlation structure supports the biological validity of the selected features, reinforces the dominance of conservation and ensemble scores in variant interpretation, and aligns with established guidelines for evaluating missense variant impact in clinical genomics.

**FIGURE 8:**
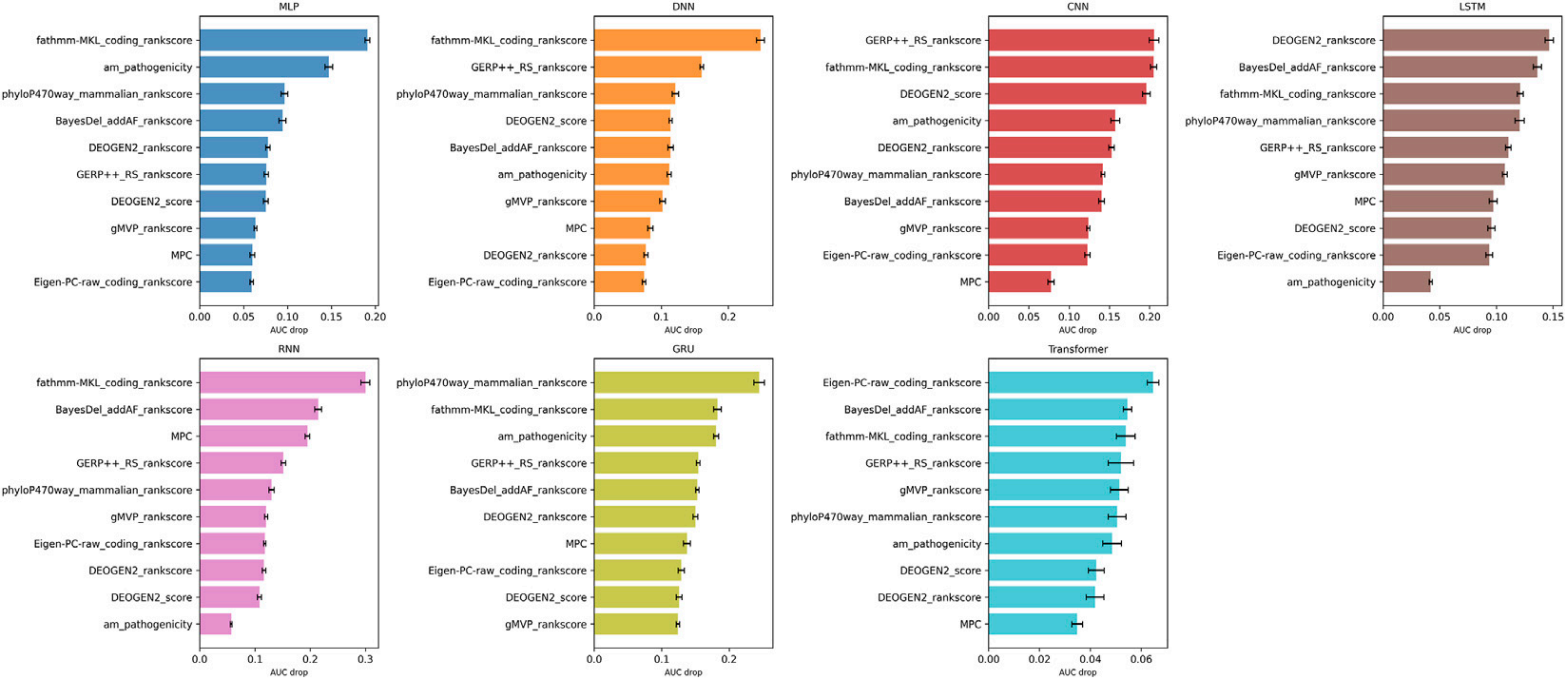
Permutation feature importance for all seven models, measured as mean AUC drop (±SD) after feature permutation on the internal test set. fathmm-MKL_coding_rankscore is the top contributor for MLP, DNN, and RNN; phyloP470way_mammalian_rankscore leads for GRU; GERP++_RS_rankscore dominates CNN; DEOGEN2_rankscore leads for LSTM; Eigen-PC-raw_coding_rankscore leads for the Transformer. Architecture-specific prioritization reflects differing internal representations across feedforward, convolutional, and recurrent models.

For interpretability, PMI and LIME were conducted. The PMI analysis was conducted to evaluate the relative contribution of each input feature to the predictive performance of all deep learning models. The results shown in Figure 8, reveal distinct prioritization patterns across models, though several features consistently emerged as highly influential. fathmm-MKL_coding_rankscore ranked first for MLP, DNN, and RNN; phyloP470way_mammalian_rankscore led for GRU; GERP++_RS_rankscore dominated CNN; DEOGEN2_rankscore led for LSTM; and Eigen-PC-raw_coding_rankscore led for the Transformer. Conservation scores, namely phyloP470way_mammalian_rankscore, Eigen-PC-raw_coding_rankscore, and GERP++_RS_rankscore, were recurrently identified among the top contributors across architectures, indicating their relevance in capturing sequence conservation and evolutionary signals. Models like LSTM and GRU placed greater importance on conservation-based features, consistent with their sequential processing capabilities, which likely exploit position-sensitive feature interactions within the ordered input vector. Meanwhile, CNN and DNN models exhibited a broader reliance on scoring metrics such as FATHMM and BayesDel_addAF, which align with their spatial abstraction strengths. Notably, am_pathogenicity and MPC ranked lower overall yet demonstrated moderate importance in certain architectures, reflecting model-specific sensitivity to constraint and impact scores that complement the dominant conservation signals. These findings collectively underscore that while some features (Eigen-PC, phyloP) are universally valuable, deep learning models vary in how they internalize and prioritize information, depending on their architectural mechanisms for pattern extraction.

**FIGURE 9:**
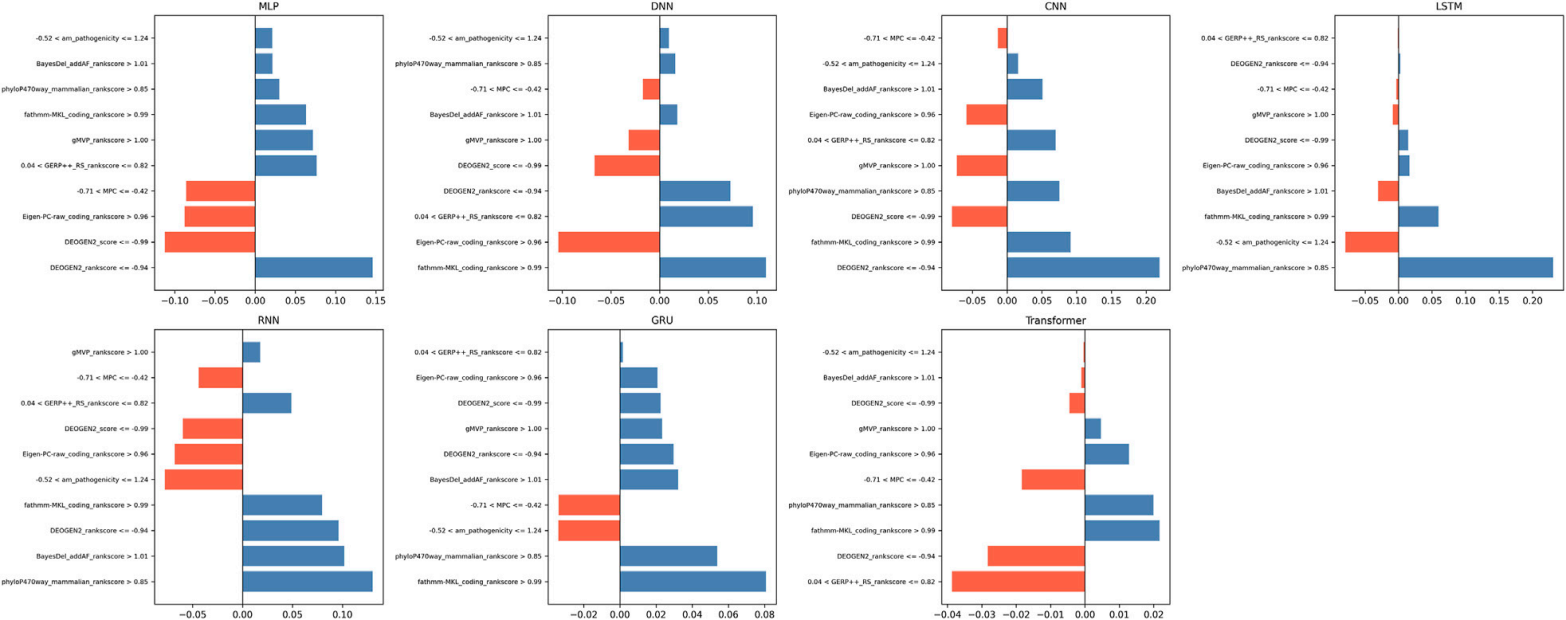
LIME explanations for a representative pathogenic variant across all seven models. Blue bars = positive contribution toward pathogenic prediction; red bars = negative. fathmm-MKL_coding_rankscore and phyloP470way_mammalian_rankscore are the strongest positive contributors across most models, consistent with Figure 8. am_pathogenicity shows dual behavior, positive in MLP and DNN, negative in CNN and GRU, reflecting architecture-specific feature interactions.

**FIGURE 10:**
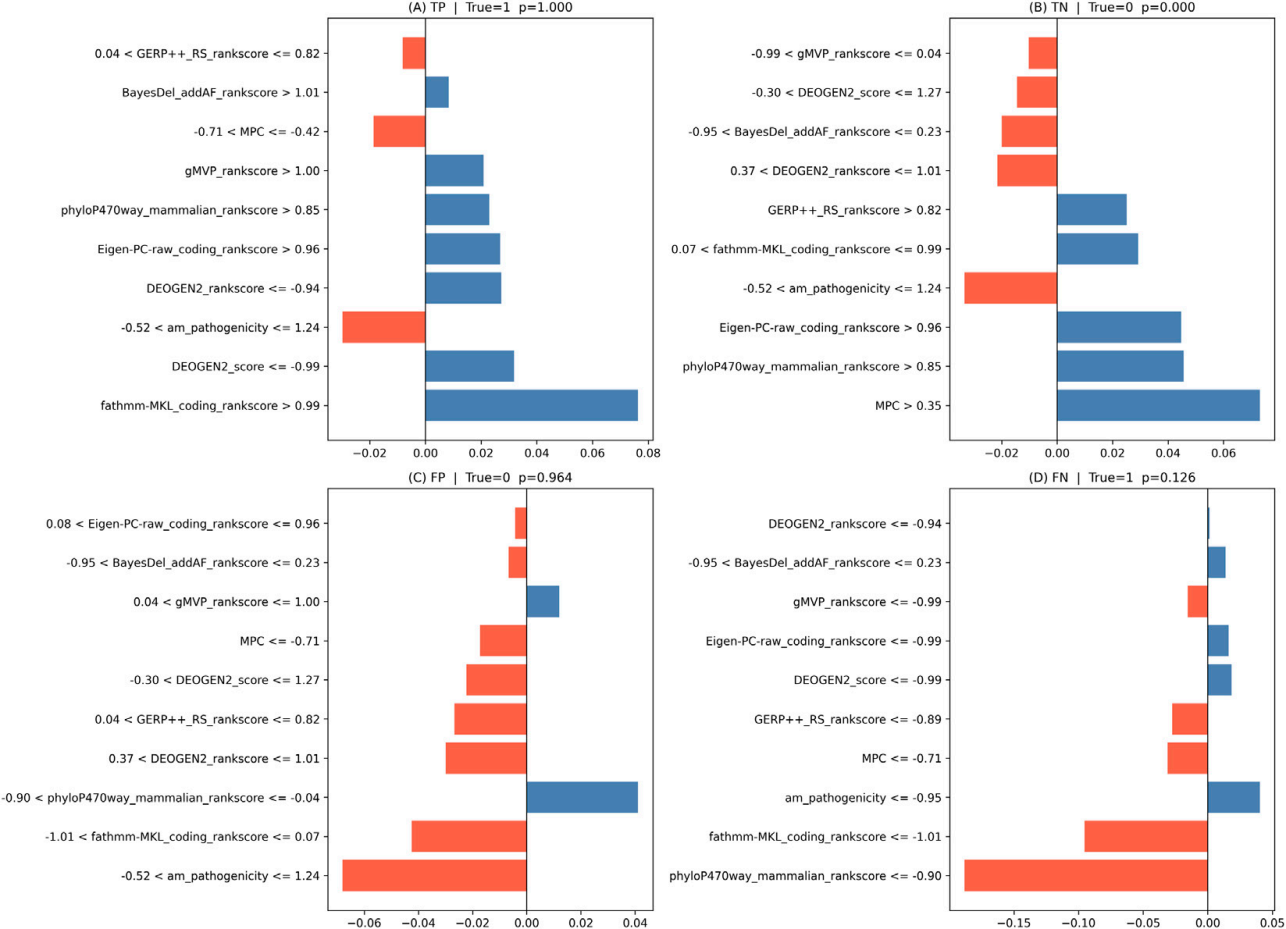
Case-level LIME explanations from the GRU model for **(A)** True Positive (p = 1.000), **(B)** True Negative (p = 0.000), **(C)** False Positive (p = 0.964), and **(D)** False Negative (p = 0.126). Blue = positive contribution toward pathogenic prediction; red = negative. The TP is driven by fathmm-MKL_coding_rankscore > 0.99; the TN by MPC > 0.35 and phyloP470way_mammalian_rankscore > 0.85. The FN is characterized by uniformly low deleteriousness signals across multiple features, including phyloP470way_mammalian_rankscore ≤ −0.90 and fathmm-MKL_coding_rankscore ≤ −1.01.

To further elucidate the decision-making rationale of each deep learning model, LIME were generated for representative predictions across the seven architectures. LIME explanations provided model-specific visualizations of the top features contributing positively (green) or negatively (red) to the pathogenicity prediction of missense variants as shown in Figure 9. fathmm-MKL_coding_rankscore and phyloP470way_mammalian_rankscore were consistently the strongest positive contributors across most models. Features like MPC, am_pathogenicity, and BayesDel_addAF_rankscore also appeared frequently in the top LIME explanations, consistent with their PMI rankings. Sequence models such as RNN, LSTM, and GRU displayed a distinct emphasis on evolutionary and conservation-based features. In particular, phyloP470way_mammalian_rankscore, and Eigen-PC-raw_coding_rankscore repeatedly influenced predictions in both LIME and PMI analyses for these architectures. This reflects the capacity of these models to weight position-sensitive feature interactions across the ordered input vector. Interestingly, am_pathogenicity > 1.24 exhibited both positive and negative contributions depending on the model, suggesting potential interactions with other feature contexts that vary by architecture. For instance, it had a negative contribution in Transformer, LSTM, and CNN, but a positive effect in MLP and DNN. This dual behavior highlights the complexity of its role and supports the need for interpretability tools like LIME to complement feature importance metrics. Overall, the high concordance between LIME and PMI results reinforces the robustness of the models’ internal reasoning and confirms that certain features, especially fathmm-MKL_coding_rankscore, MPC, and conservation-based scores, are central to accurate pathogenicity prediction regardless of the network structure. To further investigate interpretability, LIME visualizations were generated for one representative True Positive (TP), True Negative (TN), False Positive (FP), and False Negative (FN) prediction from the GRU model. As shown in Figure 10, fathmm-MKL_coding_rankscore, am_pathogenicity, and conservation features (phyloP) consistently contributed to correct predictions, while misclassifications often involved low-weight or ambiguous feature signals.

## Discussion

**FIGURE 11:**
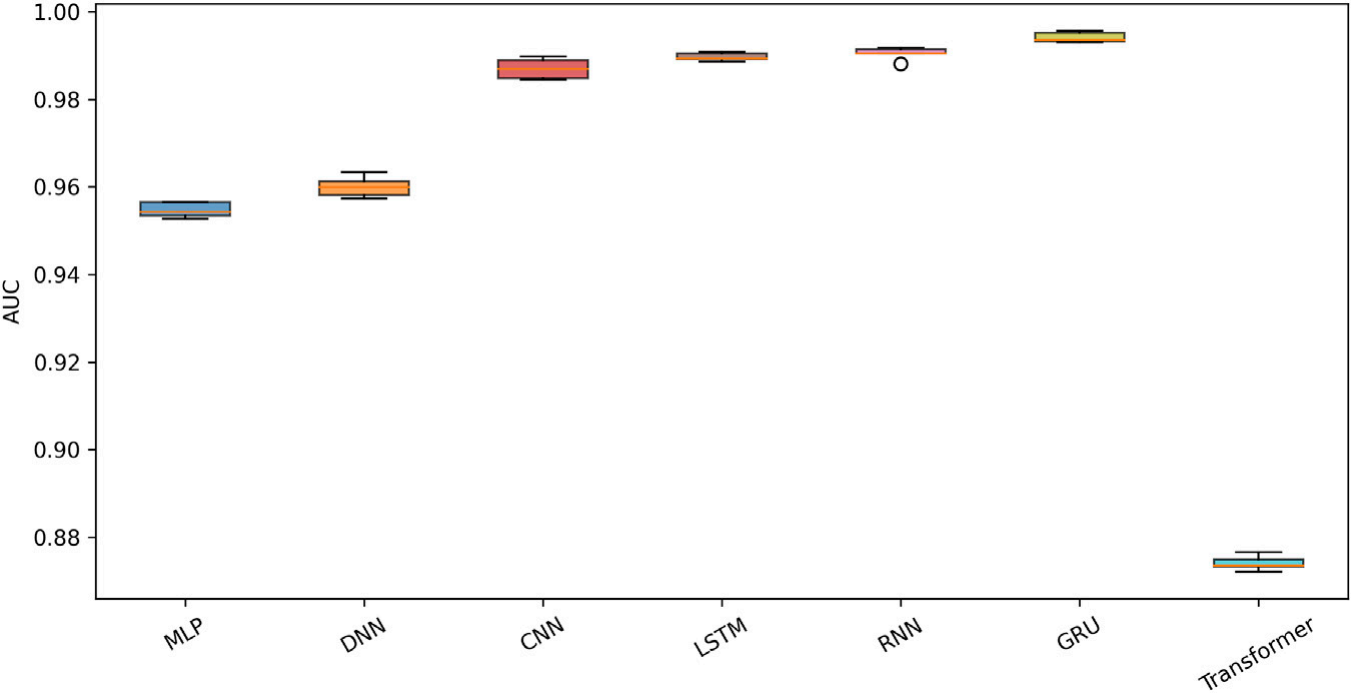
AUC distribution across five random seeds per model. GRU achieves the highest mean AUC (0.9941 ± 0.0011) with the narrowest spread. The Transformer is consistently the weakest (0.8741 ± 0.0015). These distributions support mean ± SD as the primary performance estimator. Per-seed values are in Table 2.

This study demonstrates the potential of DL models to accurately classify breast cancer missense variants as pathogenic or benign using a robust, multi-phase pipeline. Among the seven architectures tested, GRU and LSTM models outperformed others across nearly all evaluation metrics. These results align with the biological premise that missense variants carry positional dependencies, here referring to amino acid position within the protein and local sequence context, not chromosomal coordinates, which were dropped as non-informative, alongside nuanced conservation signals, attributes best captured by recurrent architectures designed for sequence modeling. Sensitivity to random seed initialization is a genuine evaluation concern in deep learning. Each model was trained across five seeds; mean AUC ± SD across seeds is reported as the primary performance estimate, avoiding optimistic bias from best-seed selection. GRU achieved the highest mean AUC (0.9941 ± 0.0011) with the narrowest spread across seeds (Figure 11; Table 2), confirming that its superiority is distributional rather than seed-specific. The best-seed run was retained only for LIME and PMI, where a single fixed prediction set is required. The narrow SD across seeds for GRU and LSTM, and the wider spread for the Transformer (0.8741 ± 0.0015), are themselves informative about architectural stability under random initialization. The superior performance of GRU and LSTM models can be attributed to their architecture’s proficiency in capturing sequential dependencies within genomic data. These models are designed to retain information over long sequences, allowing them to effectively model the positional and contextual relationships inherent in nucleotide sequences (Cahuantzi et al., 2023). Their high F1 Scores (>0.95) and MCC values (>0.91) underscore their capacity to preserve contextual relationships in complex biological inputs. These models exhibited strong discriminative power with minimal false positives on the internal test set. On the independent external set (n = 437), LSTM achieved AUC 0.9457, exceeding all eleven standalone predictors benchmarked on the same variants (Table 8), providing a first indication of generalization beyond the training cohort. Furthermore, sensitivity above 0.91 is particularly critical in a clinical genomics context, where failure to identify a true pathogenic variant could have significant consequences for diagnosis and treatment. This performance is consistent with previous findings by (Kang et al., 2023), who demonstrated that deep learning frameworks integrating sequential modeling outperformed traditional models like SIFT and PolyPhen in classifying rare BRCA1/2 missense variants. Similarly, Dutta and his team showed that stacked GRU-LSTM architectures provided significant accuracy improvements in breast cancer classification tasks, particularly for imbalanced datasets (Dutta et al., 2020). While models like AlphaMissense and REVEL provide powerful variant scoring, they often lack explainability or fail to generalize to under-represented variants of uncertain significance (VUS), reinforcing the need for interpretable models such as those proposed here. Structure-based predictors including ESM1b and EVE were not included in the external benchmark because their scores were absent or sparse in the external VEP annotation file; this is noted as a limitation and a direction for future comparison on a purpose-annotated external set. A major contribution of this work is the integration of 95% confidence intervals (CIs) for all key performance metrics, calculated using bootstrapping with 1000 iterations. This statistical enhancement allows for a more robust comparison between models and adds credibility to the reported AUCs and other metrics, ensuring that observed performance is not due to random variance. From an interpretability standpoint, the combination of PMI and LIME provided valuable insights into the decision rationale of each model. The consistent prioritization of conservation-based features such as fathmm-MKL_coding_rankscore, phyloP470way_mammalian_rankscore, and Eigen-PC-raw_coding_rankscore aligns with ACMG/AMP guidelines (Richards et al., 2015) and confirms the biological plausibility of the learned model behavior. Notably, the dual role of features such as am_pathogenicity across models (positive in MLP, negative in Transformer) underscores the complexity of feature interactions and highlights the importance of architecture-specific interpretability. To further demystify the “black-box” nature of DL, we applied LIME to four clinically relevant case scenarios, one TP, one TN, one FP, and one FN. These individual-level explanations revealed the most influential features driving the model’s predictions for each case, allowing domain experts to trace decision logic and assess biological plausibility. This is especially critical when deploying AI tools in clinical settings, where explainability can inform trust, review, and second-opinion workflows. The use of VAEs for imputing missing values represents an innovative approach in clinical genomics. Traditional imputation methods such as mean or KNN often fail to account for latent structures in high-dimensional datasets. In contrast, VAEs, as demonstrated by Qiu’s group, effectively model the data distribution to generate plausible missing values, improving robustness in downstream classification tasks (Qiu et al., 2020). By filtering features via RFE and preserving the near-equal class distribution through stratified splitting, the pipeline ensured informative learning across all models without introducing synthetic noise. This methodological rigor was complemented by statistical tests on per-variant score distributions (Shapiro-Wilk, Levene, Z-test, ANOVA), which characterize score-level properties rather than fold-level ranking stability. A related methodological concern is predictor circularity. Several RFE-selected features, namely, am_pathogenicity, fathmm-MKL_coding_rankscore, BayesDel_addAF_rankscore, and DEOGEN2 scores, are themselves trained classifiers whose training sets overlap with ClinVar, the same source used for ground-truth labels here. This constitutes partial circular input: the model may partly learn to reproduce label assignments already encoded in its features rather than discovering independent biological signal. We address this in two ways. First, ClinPred was excluded entirely before RFE because it is a direct meta-predictor trained on the same ClinVar labels used as ground truth, constituting unambiguous circular input (Table 1). Second, am_pathogenicity carries a partial circularity flag (Table 1); a gene-held-out ablation omitting am_pathogenicity yields internal AUC 0.815 ± 0.112, confirming the model retains meaningful discriminative power beyond label reproduction. The remaining features, MPC, GERP++_RS, phyloP470way, Eigen-PC, are conservation and constraint scores with no ClinVar training dependency. Circularity therefore partially inflates internal metrics; the external AUC for the best model (LSTM 0.946, Table 6) and the gene-held-out AUC (0.815) together bracket the range of performance on truly independent data. To statistically validate inter-model performance differences, we conducted Z-tests, Shapiro-Wilk tests for normality, Levene’s test for variance homogeneity, and one-way ANOVA. While Shapiro-Wilk confirmed non-normal distributions across models (p < 0.01), Levene’s test showed GRU exhibited the smallest variance deviation among all models (statistic = 13.86), far below the Transformer (3,398) and MLP (1,239), confirming its prediction score distribution is the most stable. DeLong’s test showed GRU differed significantly from LSTM (p < 0.001; Table 3), confirming their AUC difference is statistically detectable, with GRU maintaining the superior position. These statistical findings support GRU as the most stable classifier in this benchmark, warranting prioritization in future prospective evaluations. The Transformer’s instability on this task reflects a structural mismatch: self-attention requires sufficient feature dimensionality to distribute attention meaningfully, and 10 tabular features do not provide that substrate. Prior work has noted that Transformers underperform simpler architectures on low-dimensional tabular data when all models are held to identical training conditions. Future benchmarks incorporating raw sequence embeddings or protein structure features, inputs that exploit the Transformer’s native strengths, may yield a fairer comparison. Calibration and discrimination are both explicit selection criteria in this study, and GRU satisfies both. Quantitative calibration metrics (Table 7) show GRU achieves the lowest raw ECE (0.0095) and Brier score (0.0131) of all seven models, not a trade-off against AUC, but a convergence of both properties in a single architecture. MLP and DNN, despite visually smoother reliability diagrams, carry higher ECE (0.0237 and 0.0216) and substantially higher Brier scores, reflecting probability compression rather than true calibration. The Transformer, which appeared visually closer to the diagonal in Figure 3C, has the worst Brier score (0.1298), a reminder that visual inspection of reliability diagrams without quantitative decomposition is unreliable. After isotonic recalibration, GRU retains the best ECE (0.0084). This finding aligns with Abas and colleagues (Abas Mohamed et al., 2025), who emphasized the need to balance accuracy with calibrated probability output in oncology AI, a standard GRU meets here on both axes. Overall, these insights reflect a broader paradigm shift in clinical machine learning, from optimizing for accuracy alone to developing trustworthy, calibrated, and explainable AI systems.

Nonetheless, this study is not without limitations. First, the dataset integrates both germline variants (ClinVar, HGMD, BRCAExchange) and somatic variants (COSMIC, cBioPortal, TCGA) across a 105-gene breast cancer panel (Supplementary Table S1). Pathogenicity interpretation can differ between germline and somatic contexts, a germline *BRCA1* variant classified as pathogenic for hereditary breast cancer risk is not directly equivalent to a somatic mutation in the same position in a tumor sample. The current framework does not stratify predictions by variant origin, and performance within each stratum has not been separately evaluated. This is a genuine scope limitation that future work should address through origin-stratified modeling. Additionally, variant annotations are subject to database inconsistencies and periodic reclassification. Second, although the class distribution was near-balanced (11,542 pathogenic/12,498 benign, ratio ∼1:1.08), no resampling was applied; performance on datasets with more extreme imbalance warrants separate evaluation. Third, the external test set (n = 437) provides an initial generalization signal, LSTM AUC 0.9457, exceeding all standalone predictors, but it derives from the same VEP annotation pipeline and overlapping databases as the training cohort. Validation on an entirely independent cohort, ideally from a distinct population and annotation source, is required before clinical deployment can be considered (Topol, 2019). Finally, the current framework does not account for multi-omics integration or familial segregation patterns, both of which could enhance predictive accuracy.

## Conclusion

This study presents a comprehensive benchmark of deep learning architectures for breast cancer missense variant classification, establishing GRU and LSTM as the strongest performers across internal and external evaluation sets. Leveraging curated datasets, advanced imputation techniques, and rigorous feature selection, the pipeline achieved high classification performance, robust statistical validation, and interpretable outputs through LIME and permutation importance. The findings highlight the critical role of conservation-based features, such as phyloP470way, fathmm-MKL, and Eigen-PC, in driving accurate variant classification, and demonstrate that recurrent architectures are particularly well-suited to genomic data due to their ability to capture sequential dependencies. Moreover, isotonic recalibration was applied to all models and reported alongside raw metrics (Table 7; Figure 4); GRU retained the best calibration before and after recalibration (raw ECE 0.0095, post-recalibration ECE 0.0084), resolving any tension between its discriminative and probability-reliability performance. By integrating statistical assessments, interpretability, and clinically meaningful performance metrics, this work contributes a reproducible and extensible framework for applying deep learning in precision oncology. The methodology is not only applicable to breast cancer but can also be adapted to other genetic disorders where variant interpretation is crucial. Ultimately, this study demonstrates the tangible potential of deep learning to support genomic medicine, offering scalable, interpretable, and high-performance solutions for variant pathogenicity classification in breast cancer and beyond. It also provides an interpretable AI framework whose generalization to independent cohorts and prospective clinical utility remain directions for future work. Future work should prioritize three directions: origin-stratified modeling to separate germline predisposition from somatic tumor variants, external validation on independent MENA and underrepresented population cohorts, and integration of multi-omics inputs, including RNA expression and protein structural data, to expand the feature space beyond VEP-derived annotations.

## Data Availability

Datasets prepared and used in this research were downloaded from online databases including COSMIC (https://cancer.sanger.ac.uk/cosmic), CBioPortal (https://www.cbioportal.org/datasets), BRCAExchange (https://brcaexchange.org/), TCGA (https://www.cancer.gov/ccg/research/genome-sequencing/tcga), ClinVar (https://www.ncbi.nlm.nih.gov/clinvar/), HGMD Professional 2024.3 (https://www.hgmd.cf.ac.uk/), and the Catalogue for Transmission Genetics in Arabs (CTGA; https://www.cags.org.ae/ctga/).The final data structure used in this research can be available upon request from the authors. The final dataset is not publicly available due to the inclusion of data from the HGMD Professional version 2024.3 which is subscription based. A sample of the dataset can be available from the authors on reasonable request. The code used to generate the results is available on https://github.com/rahafahmad89/DL-clinically-interpretable-framework.
