# Supplementary material for: Clinically interpretable deep learning for breast cancer missense variant pathogenicity prediction
Source: Front Bioinform. 2026 Jul 21;6:1844071. doi: 10.3389/fbinf.2026.1844071 (PMC13433856; doi:10.3389/fbinf.2026.1844071)
Supplement: Supplementary file 1 [file Table1.docx]

| **Gene** | **Gene** | **Gene** |
| --- | --- | --- |
| **TP53** | **BRIP1** | **NRAS** |
| **BRCA2** | **FANCM** | **PARP2** |
| **BRCA1** | **FBXW7** | **POLD1** |
| **ERBB2** | **NBN** | **RAD51D** |
| **ATM** | **RB1** | **SETBP1** |
| **PTEN** | **ERBB3** | **ARPC1B** |
| **CDH1** | **RINT1** | **GEN1** |
| **MSH6** | **SLX4** | **PTPRF** |
| **MSH2** | **RET** | **SYNE2** |
| **CHEK2** | **IDH1** | **ALCAM** |
| **CTNNA1** | **MUTYH** | **ALK** |
| **PIK3CA** | **NTHL1** | **CDC73** |
| **NF1** | **PPM1D** | **FANCI** |
| **PMS1** | **RHOA** | **FGFR4** |
| **MLH1** | **BMPR1A** | **RECQL** |
| **BARD1** | **KRAS** | **SHBG** |
| **PALB2** | **POLE** | **VHL** |
| **BLM** | **EPCAM** | **FANCC** |
| **FANCD2** | **PALLD** | **FLNC** |
| **RAD51C** | **NF2** | **MED12** |
| **ESR1** | **CDKN1A** | **RAD51** |
| **SMARCA4** | **PDGFRA** | **SF3B1** |
| **APC** | **WRAP53** | **CDKN2A** |
| **GNAS** | **CASP8** | **CPS1** |
| **STK11** | **SDHA** | **DIS3L2** |
| **HRAS** | **ATXN7** | **EIF2B5** |
| **RAD51B** | **CACNA2D3** | **ERCC5** |
| **MET** | **DCLRE1B** | **FGFR3** |
| **PMS2** | **FAM175A** | **GPC3** |
| **EGFR** | **MTOR** | **JAK2** |
| **RAD50** | **PPP2R1A** | **KIT** |
| **CDK12** | **SMAD4** | **MRE11A** |
| **MAP3K1** | **SUFU** | **MYO7A** |
| **TSC2** | **WRN** | **PTPN11** |
| **RAD54L** | **GALNT12** | **XRCC2** |

Supplementary table S1: The list of genes included in the dataset.
